# A Smartphone-Assisted Schirmer Strip for Rapid Dual Colorimetric Sensing of Tear Glucose and pH Level

**DOI:** 10.1021/acsomega.6c10289

**Published:** 2026-10-13

**Authors:** Zekeriya Cetinkaya, Nimet Temur, Umut Gorkem Kocabas, Sadik Kucukgunay, Humeyra Aslaner, Ismail Ocsoy

**Affiliations:** † Department of Ophthalmology, 652466 Kayseri State Hospital, Kayseri 38010, Turkey; ‡ Department of Analytical Chemistry, Faculty of Pharmacy, 52958 Erciyes University, Kayseri 38039, Turkey; § Biomedical Engineering, Faculty of Electrical and Electronics Engineering, 52999 Yıldız Technical University, Esenler, Istanbul 34220, Turkey; ∥ Department of Medical Pharmacology, Faculty of Medicine, Kirsehir Ahi Evran University, Kirsehir 40100, Turkey; ⊥ Department of Family Medicine, Kayseri Faculty of Medicine, 668479 University of Health Sciences, Kayseri 38039, Turkey

## Abstract

Although tear biomarkers are routinely determined using various clinical methods, most current techniques are complex, time-consuming, and require expensive equipment. Therefore, point-of-care (POC) testing is attracting considerable attention for rapid and noninvasive analyses. Here, we present a dual-function colorimetric Schirmer strip for the simultaneous determination of tear glucose and pH using the naked eye and image processing software. In our approach, horseradish peroxidase-glucose oxidase nanoflowers (HRP–GOx@NF) are used for glucose detection, and red cabbage-derived anthocyanins are used as a pH-sensitive, nontoxic indicator. In glucose detection, the catalytic stepwise reaction of HRP–GOx@NF produced a distinct blue color within 30 s by enabling the rapid oxidation of 3,3′,5,5′-tetramethylbenzidine (TMB) in the presence of glucose, and this optimized platform exhibited a visual detection limit of 0.5 mM. Additionally, the anthocyanin-modified Schirmer strip simultaneously displayed distinct color transitions in response to varying pH values of the artificial tear fluid. This work presents a novel multiparametric paper platform that uniquely upgrades standard Schirmer strips for simultaneous, single-step tear collection, glucose sensing, and pH mapping without altering fluid flow. Like this, it demonstrates a rapid, cost-effective, and easy-to-prepare diagnostic tool offering significant advantages over conventional methods.

## Graphical abstract

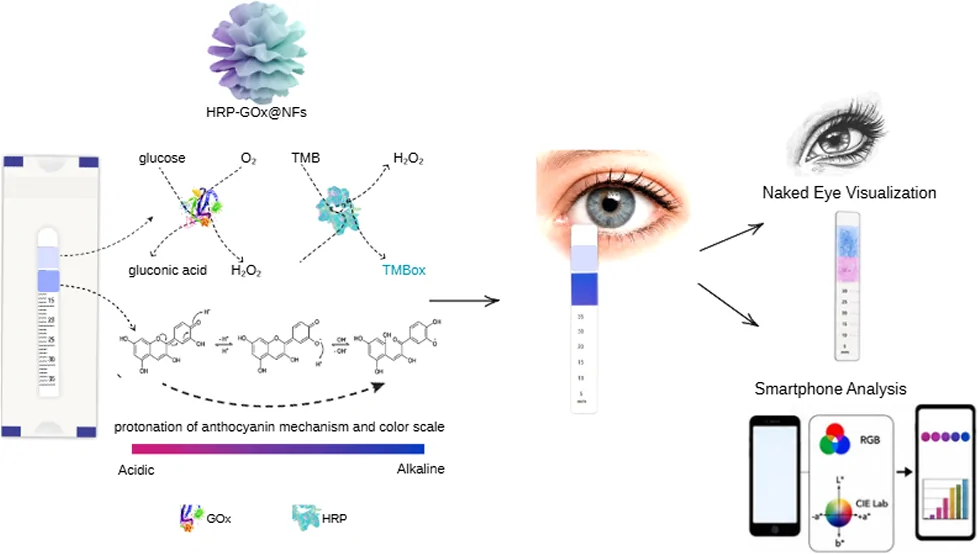

## 1 Introduction

The increasing interest in noninvasive and point-of-care (POC) diagnostic methods has driven research into the use of biological fluids as alternatives to conventional blood sampling. In this context, tear fluid has emerged as a promising diagnostic medium due to its safe, noninvasive, and repeatable collection and its ability to provide access to a wide range of biomarkers associated with both ocular and systemic health.^1,2^ The Schirmer test is one of the most frequently employed clinical methodologies for the collection of tears. It is always used to evaluate tear secretion in patients with dry eye disease. Beyond its conventional clinical use, the Schirmer strip has gained increasing attention as a paper-based analytical platform, as it enables tear collection and subsequent biomarker analysis in a simple and portable format.^3^

Among the various tear biomarkers, glucose has received particular attention due to its close relationship with systemic glucose metabolism. Diabetes mellitus is a chronic metabolic disorder that requires regular monitoring to prevent severe complications. Despite the long-established role of self-monitoring of blood glucose as a management strategy for diabetes, conventional methods remain invasive, costly, and offer only intermittent measurements. Consequently, tears have been investigated as a potential biological fluid for glucose monitoring due to their physiological composition, which reflects an individual’s metabolic status. In particular, tear glucose has attracted considerable interest as a potential noninvasive alternative for glucose monitoring. Recent advances in biosensing technologies, including highly sensitive electrochemical and wearable sensing platforms, have further improved the feasibility of glucose detection in tear samples.^4,5^ In addition to glucose, tear pH is a significant physicochemical parameter associated with ocular surface health and tear film homeostasis. The tear film is a complex biological fluid containing various biochemical components, including electrolytes, metabolites, proteins, and lipids. Alterations in its composition may therefore reflect changes in ocular surface conditions. Recent advances in colorimetric sensing platforms have demonstrated the feasibility of noninvasive tear pH monitoring, including wearable devices capable of simultaneous detection of multiple tear biomarkers. This highlights the potential of tear pH analysis for POC ocular health assessment. Consequently, simultaneous monitoring of tear glucose and pH levels may provide complementary information for evaluating metabolic status and ocular surface conditions.^6,7^

The utilization of paper-based colorimetric sensors has attracted increasing attention for POC applications. This growing interest can be attributed to several factors, including their simplicity, cost-effectiveness, portability, and rapid visual response. The integration of smartphone-based image analysis further enhances their quantitative performance by enabling objective color assessment through RGB analysis. To enhance the sensitivity of colorimetric biosensors and address the limitations of natural enzymes, nanozymes have been investigated as stable and cost-effective alternatives due to their excellent catalytic activity and operational stability. In particular, nanoflower (NF)-structured nanozymes offer abundant active sites and large surface areas, rendering them promising materials for signal amplification in biosensing platforms.^8–11^

Natural color indicators, particularly anthocyanin-based pigments, have gained attention as environmentally friendly alternatives for pH sensing due to their biocompatibility, low cost, and pH-responsive color scale transitions. Red cabbage-derived anthocyanins exhibit distinct color variations over a wide pH range, making them suitable for visual and smartphone-assisted colorimetric analysis.^12,13^ However, the development of simple, low-cost platforms capable of simultaneously detecting multiple tear biomarkers while preserving portability and ease of use remains challenging. The motivation of this study was to develop a simple, low-cost, and portable platform for simultaneous analysis of tear glucose and pH using a single paper-based device. The combination of tear collection, dual-analyte colorimetric sensing, and smartphone-assisted analysis was designed to provide a practical proof-of-concept approach for rapid tear-based biosensing. In this study, a rapid colorimetric Schirmer strip was developed for the simultaneous detection of tear glucose and pH by integrating NF-structured nanozymes with red cabbage anthocyanin extract (Scheme 1). In these developed tests, HRP-GOx@NFs were synthesized in situ to enable glucose detection. If glucose was present in the tear fluid flowing over the test strip, the glucose oxidase (GOx) enzyme broke it down to release hydrogen peroxide (H_2_O_2_), resulting in a colorimetric change due to the oxidation of 3,3′,5,5′-tetramethylbenzidine (TMB), which had been added to the medium beforehand, catalyzed by horseradish peroxidase (HRP). Additionally, tear pH—a critical indicator of eye infections—was determined based on the protonation of anthocyanin molecules. The rapid tests were developed to detect glucose at different concentrations, with optimized glucose selectivity. In addionatial, pH-dependent color changes were observed simultaneously. The proposed platform integrates noninvasive tear collection with portable colorimetric detection and smartphone-assisted RGB analysis, providing a cost-effective and practical approach for POC ocular health assessment and glucose monitoring.^14^

**Scheme 1:**
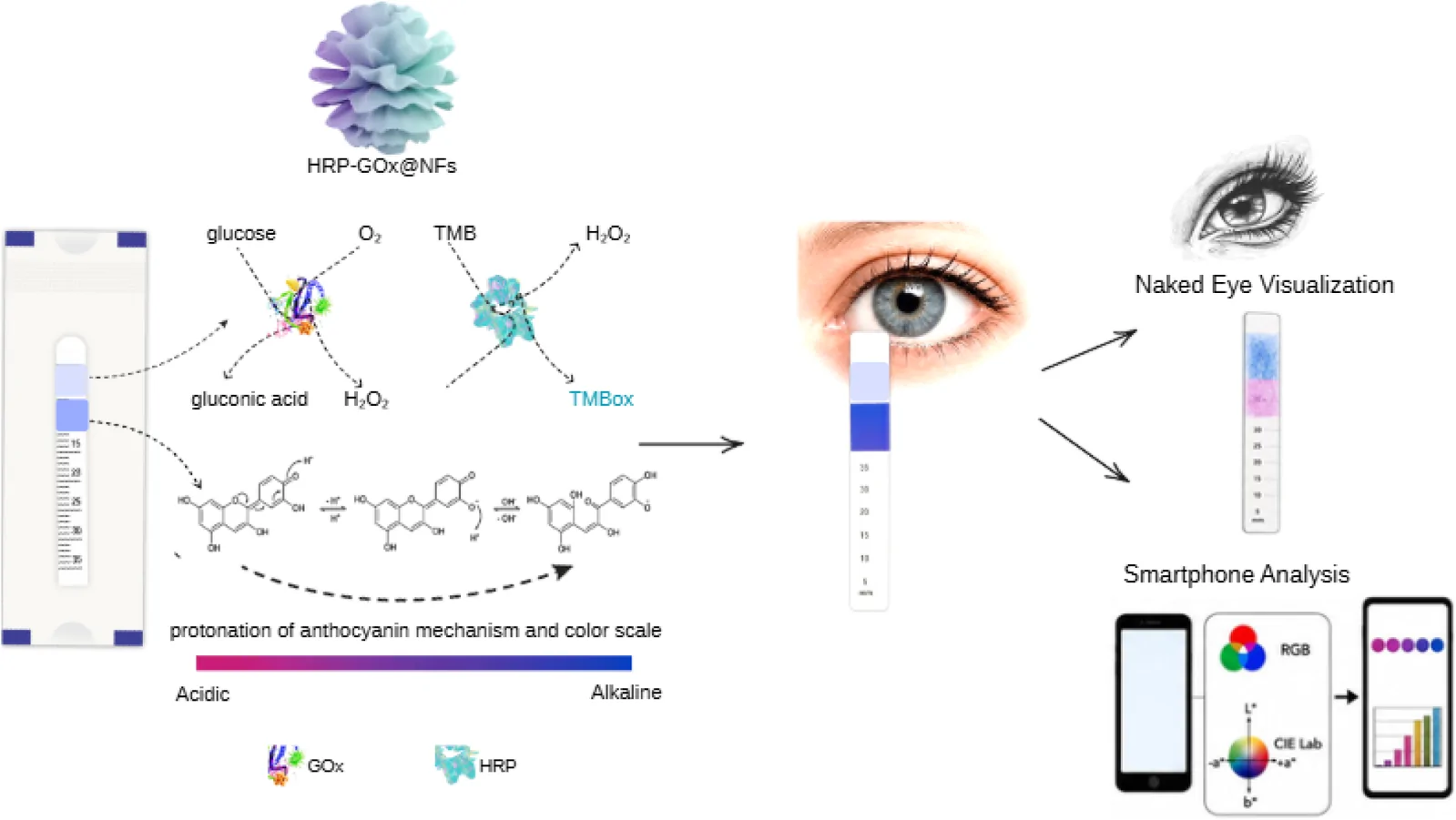
Schematic Representation of the Development of HRP-GOx@NFs and Anthocyanin-Based Colorimetric Schirmer Tests and Their Use in Glucose-pH Determination from Eye Tear^a^

a (Created with https://www.canva.com/).

## 2 Experimental Section

### 2.1 Materials

The enzymes horseradish peroxidase (HRP) and glucose oxidase (GOx), as well as copper sulfate (CuSO_4_), PBS tablets, sodium chloride, sodium bicarbonate, calcium chloride, chitosan, and 3,3′,5,5′-tetramethylbenzidine (TMB), were purchased from Sigma-Aldrich. Carboxymethylcellulose (CMC) was purchased from Akkim Kimya.

### 2.2 HRP-GOx NFs Synthesis and Characterization

The HRP-GOx@NF synthesized for glucose determination were prepared by modifying a method previously described in the literature.^15,16^ First, 0.1 mg/mL GOx and 0.02 mg/mL HRP were dissolved in PBS buffer (10 mM, pH 7.4), and then freshly prepared CuSO_4_ at a concentration of 0.8 mM was added. The resulting solution was incubated at room temperature (RT: 25 °C) for 3 days. During the synthesis process, crystallization begins between Cu^2+^ and PO_4_, forming Cu_3_(PO_4_)_2_ crystals. Subsequently, during the growth phase, HRP and GOx are incorporated into the structure, resulting in the formation of HRP-GOx-Cu_3_(PO_4_)_2_ nanopetals; the aggregation of these pellets enables the synthesis of HRP-GOx@NFs. Additionally, another synthesis was carried out on Schirmer strips. The Schirmer strips were immersed in a freshly prepared reaction solution. The formation of NFs following crystallization occurred on the strips.

The morphology and size analysis of the synthesized HRP-GOx@NFs were characterized using Scanning Electron Microscopy (SEM), elemental distribution analysis via Energy Dispersive X-ray Analysis (EDX), and functional group analysis via Fourier Transform Infrared (FTIR) techniques.

### 2.3 Artificial Tear Fluid (ATF) Preparation

Artificial tears were prepared according to a previously published method.^17,18^ First, 0.67 g of sodium chloride, 0.20 g of sodium bicarbonate and 8 mg of calcium chloride were dissolved in distilled water. The mixture was stirred at room temperature until homogeneous. The pH was then adjusted to 7.4 to make it neutral. To adjust the viscosity of the artificial tears, 3% CMC was stirred in and the solution was stored at room temperature for use in the experiments.

### 2.4 Red Cabbage (*Brassica oleracea*) Extract (RCE) Preparation

We followed the previous study for preparation of red cabbage (*Brassica oleracea* *L*.) extract.^19,20^ Briefly, red cabbage obtained from the local market was first washed and then cut into small pieces. The certain amount of small pieces were added into distilled water, then the mixture was boiled for 30 min for extraction. The purple-colored extract was cooled down to room temperature and then it was filtered through Whatman No. 1 filter paper. The filtered extract rich in anthocyanin groups was stored at 4 °C for further use.

### 2.5 Preparation of Colorimetric Schirmer Tests

The colorimetric Schirmer tests were developed by modifying kits purchased from a local medical supplier. HRP-GOx@NFs were impregnated into a specific region of the tests. The glucose detection mechanism is based on the fact that GOx breaks down glucose to release H_2_O_2_ in the presence of glucose. Under HRP catalysis, the TMB substrate is then oxidized, turning blue. To this end, a TMB solution containing 1% chitosan was prepared in an acetate buffer solution, which was then dropped onto the drying HRP-GOx@NF regions and left to dry. An RCE solution was also impregnated into another region. Preprepared ATF solutions at different pH levels and glucose concentrations were tested on test strips. After a 30-s incubation at room temperature, the release of H_2_O_2_, depending on the glucose content, triggered the oxidation of TMB catalyzed by HRP, causing the solution to turn from colorless to blue. The RCE solution, which had the normal pH of tears (pH 7.4 and a blue color), underwent protocytanization due to the different pH levels of the ATFs, resulting in a color change. The results were analyzed both visually and using a mobile app that performed RGB analysis.

### 2.6 Digital Image Processing

Tests that could be analyzed colorimetrically were photographed for digital imaging. The captured images were saved in JPEG format for analysis and imported into the ImageJ program. The ImageJ software was used to calculate RGB (Red/Green/Blue) average values and Euclidean distance (Delta E) analyses in the area where regional color changes occurred. To quantitatively determine the colorimetric change that occurred, the image pixels were separated into red, green, and blue channels, and the average values of these channels were calculated. For the color image processing analysis, the changes between the initial color and the color resulting from incubation were compared using the CIE 1976 Lab color difference method, and Delta E (ΔE) was applied. The ΔE formula is based on measuring the color differences between two images.^21,22^

1 $$\Delta E=[{\left(\Delta L\right)}^{2}+{\left(\Delta a\right)}^{2}+{\left(\Delta b\right)}^{2}{]}^{1/2}$$

The values of “L,” “a,” and “b” given in the formula in eq 1 represent the dimensions of the CIE Lab color space. The *a*-axis extends from red to green, and the *b*-axis extends from yellow to blue. The L-axis extends from black to white. Based on these parameters, the difference is low between images of similar colors, while the difference is high between images of different colors.^23,24^ This converts the limits of what the human eye can perceive into numerical data through ΔE analysis. In this study, the B/R ratio was used to indicate color changes toward blue in glucose concentration (where B increases and R decreases), while the R/B ratio was used to indicate pH changes toward pink/red (where R increases and B decreases). The RGB channel ratios were selected based on the dominant color component that increased during the reaction. The developed colorimetric Schirmer tests use the color difference to enable more accurate analysis based on glucose concentration and pH level.

#### 2.6.1 Development of a Smartphone Application

A smartphone app developed using Kotlin with image processing capabilities has been created for the quantitative analysis of colorimetric Schirmer tests. The app, which runs on Android versions 4 through 10, does not require an Ethernet connection to access the device’s camera or files. The analysis begins with a prompt in the main menu stating, “Please select the parameters as glucose or pH in the correct order.” Through this interface, the steps for capturing images and performing the analysis begin. After photographs of the modified Schirmer tests are taken, the relevant region is selected, and the RGB values and Euclidean distance are calculated for this area. As a result of the analysis, the RGB values, Euclidean distance, and the test result are displayed on the screen. In the Euclidean distance (ED) formula, values of 25 or higher, as expressed in eq 2, indicate a positive result. This confirms the presence of glucose. ED threshold values were determined to distinguish between pH changes and glucose concentrations. Consequently, a smartphone app was developed to ensure reliable and highly accurate results.^25,26^

2 $${\mathrm{ED}}^{2}={\left(R_{2}-R_{1}\right)}^{2}+{\left(G_{2}-G_{1}\right)}^{2}+{\left(B_{2}-B_{1}\right)}^{2}$$

## 3 Results and Discussion

NF synthesis occur based on coordination reactions between carboxyl or amine groups in organic molecules and metal ions. In the synthesis, Cu_2_(PO_4_)_2_ crystals first form between Cu^2+^ ions and PBS, coordinating with the functional groups (particularly amine groups) found in the structures of the HRP and GOx enzymes. In this complex, nuclei are formed for the formation of nanopetals. As the nanopetals bond hierarchically with one another, saturation is reached, and flower-like morphologies are obtained.^27^ The morphology and size of the synthesized HRP-GOx@NF particles were evaluated using various characterization methods, including elemental analysis and the identification of functional groups. The morphology of the HRP-GOx@NF particles synthesized via the postmodification method and used for impregnation onto Schirmer papers is shown in Figure 1A and 1B. Meanwhile, the morphology of the HRP-GOx@NF particles synthesized via the in situ method is shown in Figure 1C. In both NF synthesis methods, flower-like nanostructures were observed. Due to the limitations arising from the need for a greater amount of free enzyme and the requirement for homogeneity in coating all of the paper with NFs during in situ synthesis, the experiments were continued using the postmodification synthesis method. In additionally, the presence of Cu metal was confirmed by EDX analysis (Figure 1D). Quantification confirmed the presence of carbon (13.85 wt %), oxygen (19.08 wt %), sodium (28.09 wt %), phosphorus (4.27 wt %), chlorine (28.20 wt %), potassium (0.67 wt %) and copper (5.83 wt %). The C and O signals originate from the organic backbone of the encapsulated HRP and GOx enzymes, and the Cu and P signals confirm the inorganic copper phosphate scaffold of the hybrid nanoflowers. The presence of Na, Cl and trace K is attributed to residual buffer ions (PBS/saline solution) used during synthesis and washing.

**Figure 1:**
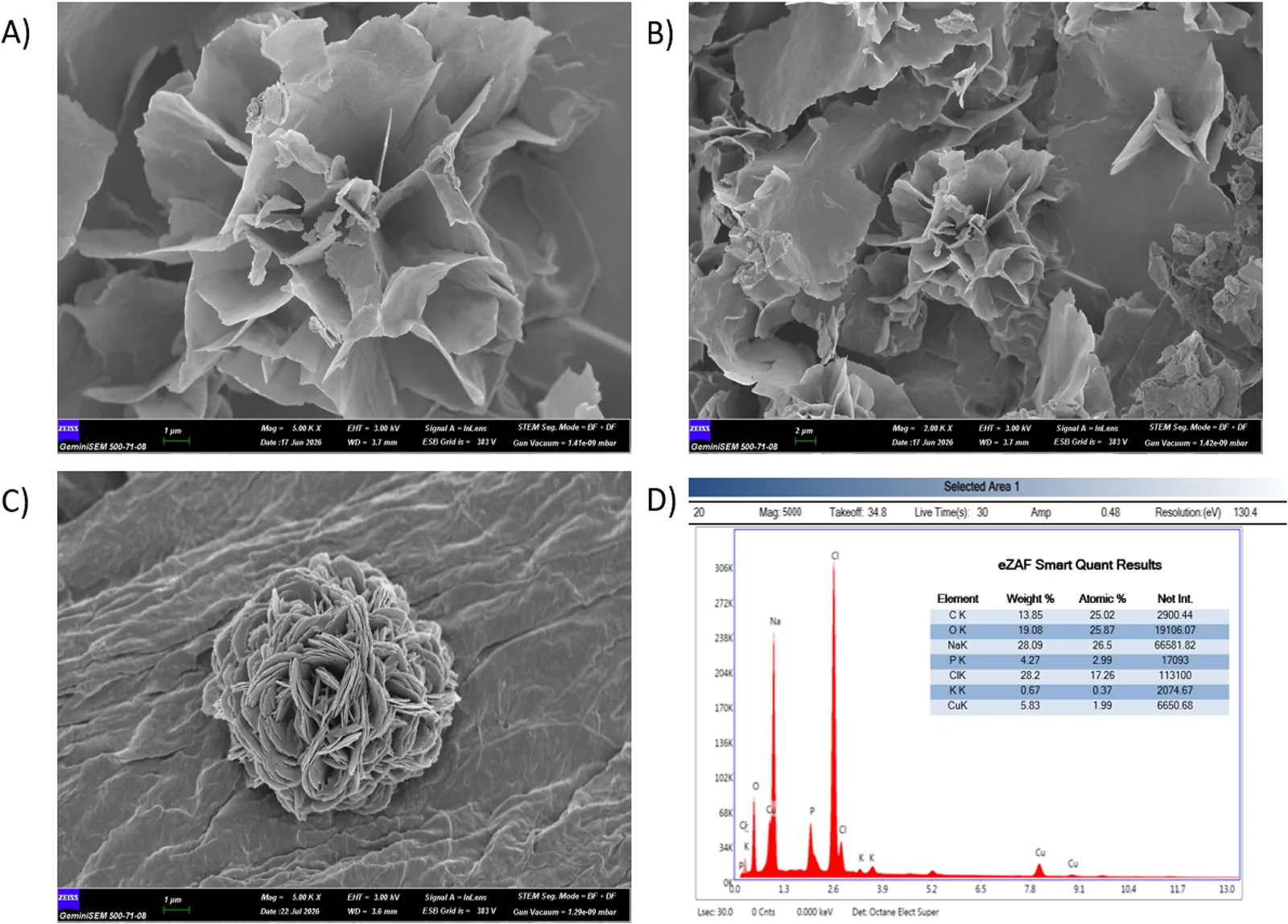
Characterization of HRP-GOx@NF: A)–B) Post modification synthesis of HRP-GOx@NF SEM images, C) in situ of HRP-GOx@NF and D) EDX analysis.

The structures of HRP-GOx@Nf were confirmed by FTIR analysis. (Figure 2) In the FTIR spectra, characteristic peaks associated with the protein/organic structure are clearly visible in both GOx and HRP in the 1638–1634 cm^–1^ (amide I) and 1517–1515 cm^–1^ (amide II) regions (Figure 2A and 2B). In the HRP-GOx@NF FTIR spectrum shown in Figure 2C, the sharp peaks in the 1079.5–987.96 cm^–1^ range confirm the presence of phosphate (PO_4_^3–^) groups that constitute the inorganic structure. Additionally, the peak at 525.97 cm^–1^ indicates metal-oxide/coordination bonding. The FTIR spectra demonstrate that the organic–inorganic hybrid NF structure has been successfully synthesized.

**Figure 2:**
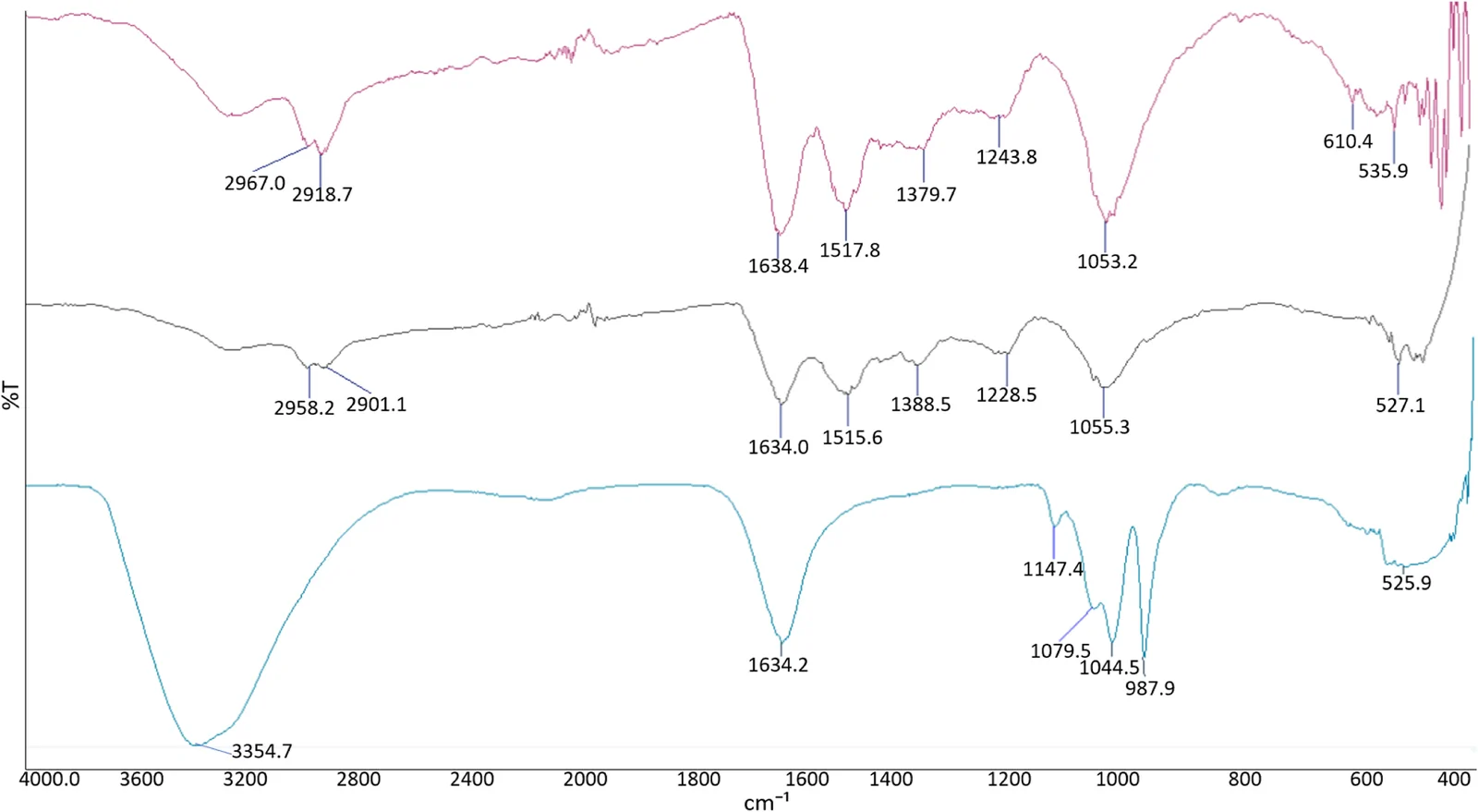
FTIR spectra: HRP (pink line), GOx (black line) and HRP-GOx@NF (blue line).

The colorimetric response performance of the Schirmer strip was studied as a function of HRP–GOx@NF concentrations (1, 2.5, 5, and 10 mg mL^–1^) for detection of 10 mM glucose in artificial tear fluid (ATF) as presented in Figure 3. While no color change was observed in the presence of 1 mg mL^–1^ HRP–GOx@NF, however blue color was slightly appeared using 2.5 mg mL^–1^ HRP–GOx@NF. In addition to that 5 mg mL^–1^ and 10 mg mL^–1^ HRP–GOx@NF resulted in blue color with the same color intensity. Then, we used 5 mg mL^–1^ HRP–GOx@NF as an ideal NF concentration for all subsequent experiments.

**Figure 3:**
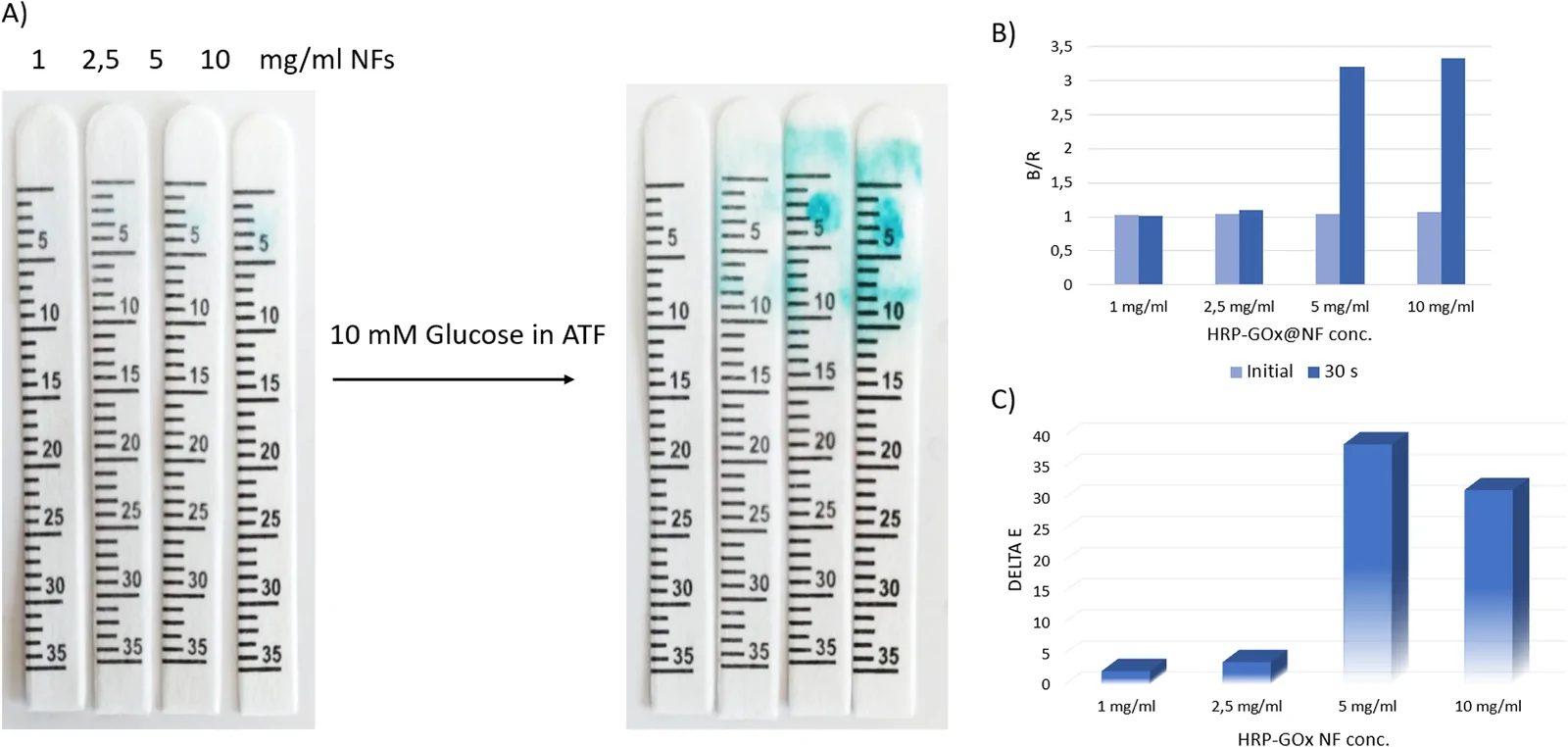
Colorimetric Schirmer strip: A) Effect of different HRP–GOx@NF concentrations on the colorimetric response of the Schirmer strip, B) RGB and C) Delta E analysis.

Nanoenzyme-based sensing systems are attracting increasing interest as an alternative to traditional enzyme-based assays due to their excellent catalytic properties and improved stability.^28–30^ In this study, HRP–GOx@NFs were used as catalytic components in a Schirmer strip-based colorimetric glucose detection platform. Optimization results showed that increasing the HRP–GOx@NF concentration increased the blue color generation, but no significant improvement was obtained when the concentration was increased from 5 to 10 mg mL^–1^ of HRP-GOx@NFs (Figure 3A). Therefore, 5 mg mL^–1^ was selected as the optimal concentration for subsequent experiments. In addition, by calculating the B/R ratios and Delta E values, it was confirmed that the 5 mg mL^–1^ of HRP-GOx@NFs concentration exhibited the best color appearance (Figure 3B and 3C). This behavior can be attributed to the catalytic properties of the synthesized HRP-GOx@NFs; where sufficient catalytic active site is needed for effective substrate conversion, while excessive NFs loading may not further improve the colorimetric signal due to limitations in substrate availability.^31,32^

The analytical performance of the developed Schirmer strip was evaluated as concentration dependent on glucose. As shown in Figure 4A, the color intensity increased progressively with increasing glucose concentration following 30 s of incubation. A distinct blue color became visible at 0.5 mM glucose, and the color intensity increased further with increasing glucose concentration. Based on naked-eye and supported with image process, the visual limit of detection the proposed assay was determined to be 0.5 mM.

**Figure 4:**
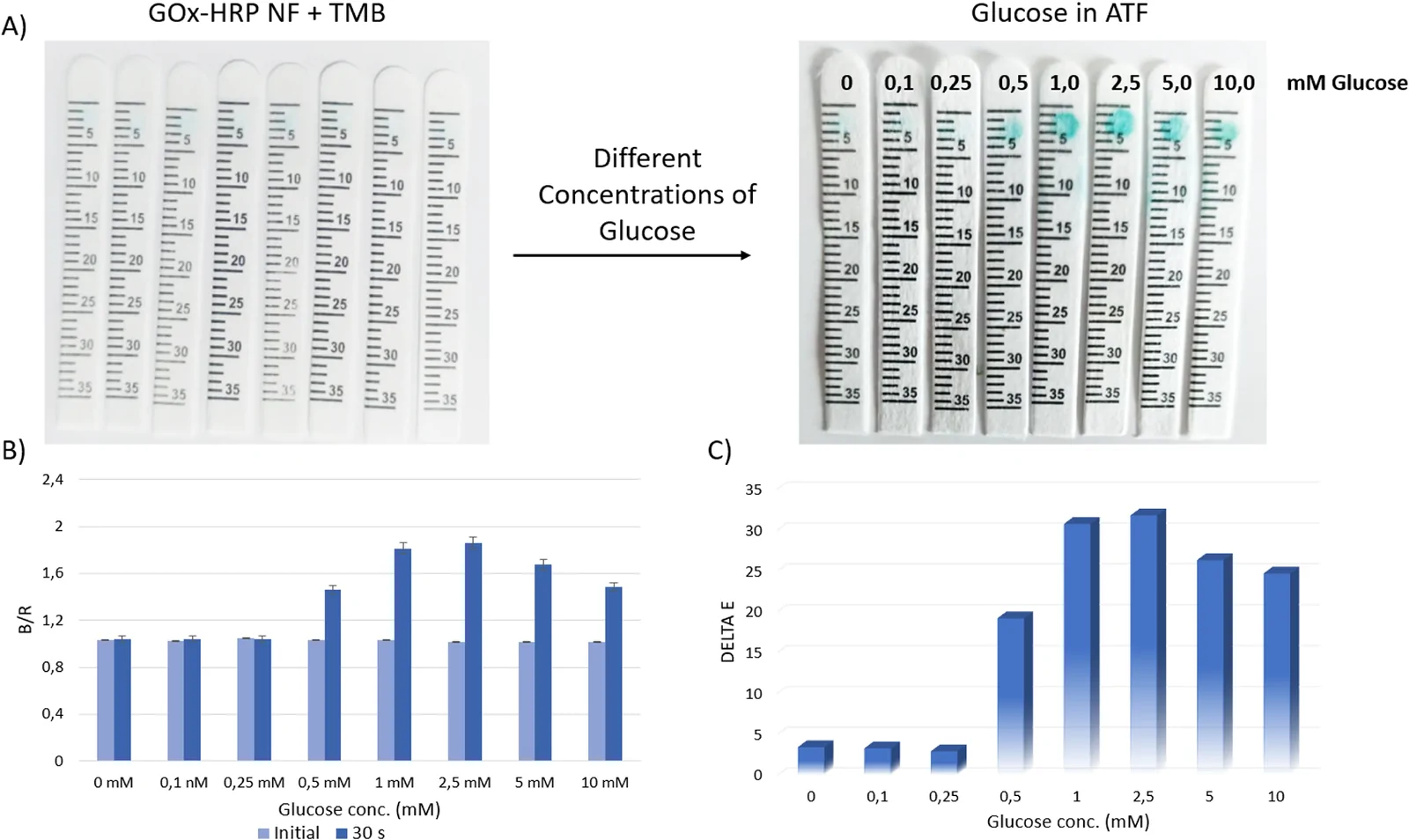
Schirmer strip toward different glucose concentrations: A) Colorimetric response, B) RGB and C) Delta E analysis, (*n* = 3).

The colorimetric changes seen in Figure 4A were confirmed by RGB and Delta analysis. The results show that there is a significant detection limit at a concentration of 0.5 mM glucose. (Figure 4B and 4C) Additionally, an analysis module based on the detection of different glucose concentrations in HRP-GOx NF synthesized via in situ synthesis and Schirmer paper tests was performed (Figure S1). Here, it was observed that the glucose detection limit of 0.5 mM was the same. Furthermore, color changes were monitored by immersing Schirmer products in solutions of different amounts of synthesized HRP-GOx NF (Figure S2). Analyses were continued with the modified method to reduce the use of free enzyme and improve stability rates. A clear color change at a level of 0.5 mM demonstrates that the sensor can clinically detect hyperglycemic limits without a device. This shows that the developed platform can be safely used in POC applications without the need for any optical reader.^7^ Moreover, we determined the quantitative analytical sensitivity of the colorimetric paper sensor by plotting a linear calibration curve of the B/R density ratio against glucose concentration. Based on IUPAC criteria and the standard deviation from triplicate blank measurements, the limit of detection (LOD) was calculated to be 0.083 mg mL^–1^ (0.42 mM). This calculation, closely aligns with the threshold of the experimentally observed visual colorimetric response (0.5 mM), confirming the strip’s high sensitivity and reliability for monitoring tear glucose.

GOx-based selective detection strategies are widely used in glucose biosensors for accurate glucose determination.^33^ In this study, the selectivity of the HRP–GOx@NF Schirmer strip was evaluated using glucose, sucrose, lactose, maltose, fructose and redox-active components that tears may contain (albumin: alb, ascorbic acid: AA, electrolytes: elect), each at a concentration of 10 mM in ATF. As shown in Figure 5A, only glucose produced a significant blue color after 30 s of incubation, no color change was observed for the other carbohydrates and redox-active components under the same experimental conditions. This result validates the specific recognition capability of GOx-based sensing sensors and demonstrates the potential of the developed Schirmer tests to selectively detect glucose. The prepared HRP-GOx@NF assemblies remained stable and exhibited activity in repeated tests, even after 60 days. In addition, in the specificity analyses, RGB and Delta analyzes performed confirm that the test is specific to glucose (Figure 5B and 5C). This high selectivity is due to the high biological specificity of the GOx enzyme toward its substrate, β-d-glucose.^34^ In enzymatic biosensor studies previously described in the literature, the stereochemical structure of the GOx enzyme’s active site specifically catalyzes β-d-glucose to d-glucono-δ-lactone (GDL) and H_2_O_2_, and does not cross-react with other carbohydrates.^35^ Furthermore, the hybrid NF structure of GOx and HRP maintained the stability of the enzymes, ensuring the continuation of high specificity. These results confirm the applicability of modified Schirmer tests that do not yield false-positive results for other components in the medium.

**Figure 5:**
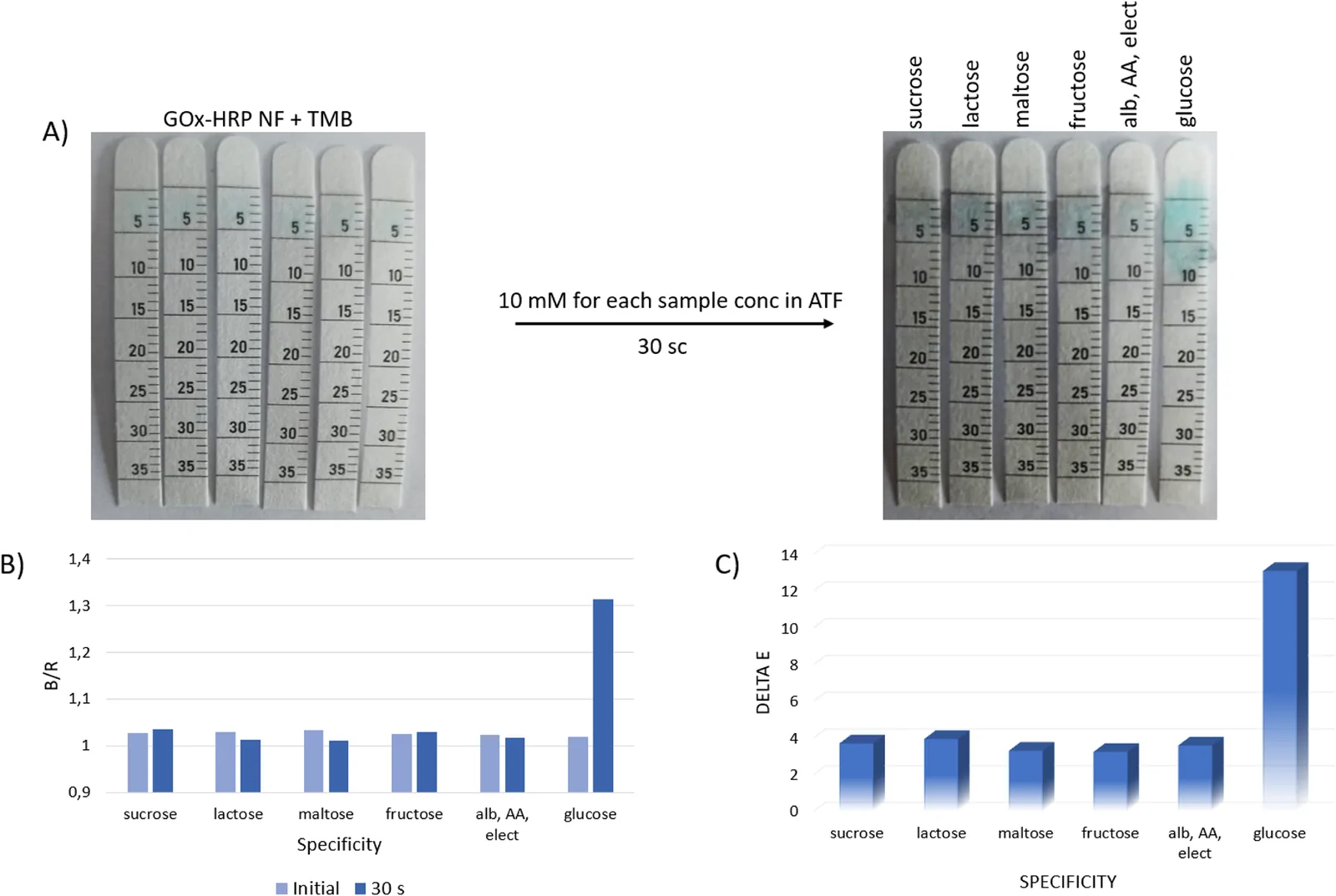
Specificity of the colorimetric assay toward glucose in comparison with other carbohydrates and redox-active component (alb, AA, elect): A) Colorimetric response, B) RGB and C) Delta E analysis.

The pH sensing ability of plant-based anthocyanin-modified Schirmer strips was investigated by observing color changes using ATF samples with varying pH values. Before running the ATF sample on the strip, it was impregnated with a 15% anthocyanin solution at pH 7.4, initially creating blue pH regions (Figure S3). To demonstrate the pink color change with pH variation, R/B ratios and Delta E values were calculated. These data show that paper-based Schirmer tests can be analyzed both visually and quantitatively (Figure 6). The colorimetric property exhibited by anthocyanins is based on the molecule’s transformation into a red-colored flavylium cation in an acidic environment, and into quinonoid base structures as the pH increases. When bacterial growth in tears, the pH level drops as a result of their activity. In this acidic environment, anthocyanin converts to the flavilium cation form, taking on a pink color. This color change enables infections to be detected visually using a simple and effective method.^14^ It is known that tear pH’s in ocular diseases, dry eye syndrome, and metabolic changes associated with diabetes.^36^ Accordingly, integrating simultaneous pH measurement on the same strip provides a more reliable and wide diagnostic enabled from a single tear sample by supporting glucose determination with a secondary physiological parameter. Despite the strip being functionalized with multiple reagents in this manner, unobstructed and continuous wicking of the test fluid has been demonstrated (Figure S4). Therefore, the traditional Schirmer test, which is used in clinical practice for the standard diagnosis of dry eye, has been combined with glucose detection and pH measurement on a single platform.

**Figure 6:**
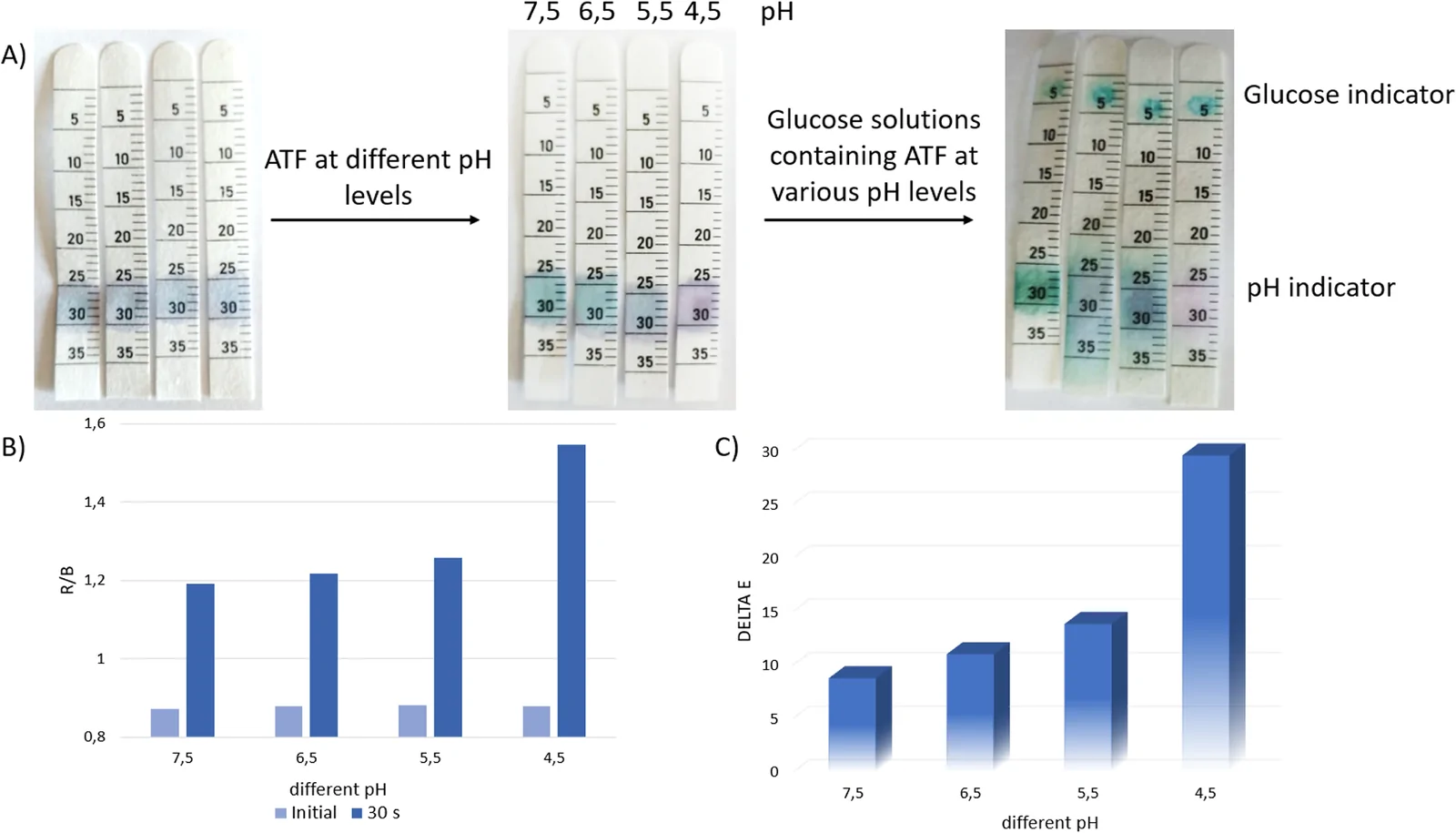
Colorimetric response of the anthocyanin-modified Schirmer strips toward artificial tear fluids with different pH values: A) Colorimetric response, B) RGB and C) Delta E analysis.

In conclusion, the HRP–GOx@NF and anthocyanin-functionalized Schirmer strip developed in this study offers a simple and portable POC strategy by combining tear collection, glucose detection, and pH analysis in a single paper-based platform.

A smartphone-based mobile app has been developed to enable easy and quantitative analysis of the colorimetric Schirmer tests prepared as shown in Figure 7. The user interfaces of the app, which we have named “Ocusense,” are shown in Figure 7, and it presents test results through a user-friendly interface. The app allows users to select analysis parameters for the prepared tests and perform the corresponding measurements. In ready-to-use paper-based modified Schirmer tests, it offers simplified quantitative analysis following a single-step tear collection procedure and a 30-s incubation period. The fact that no equipment is required and the analysis does not involve a heavy workload demonstrates the test’s potential for widespread use.

**Figure 7:**
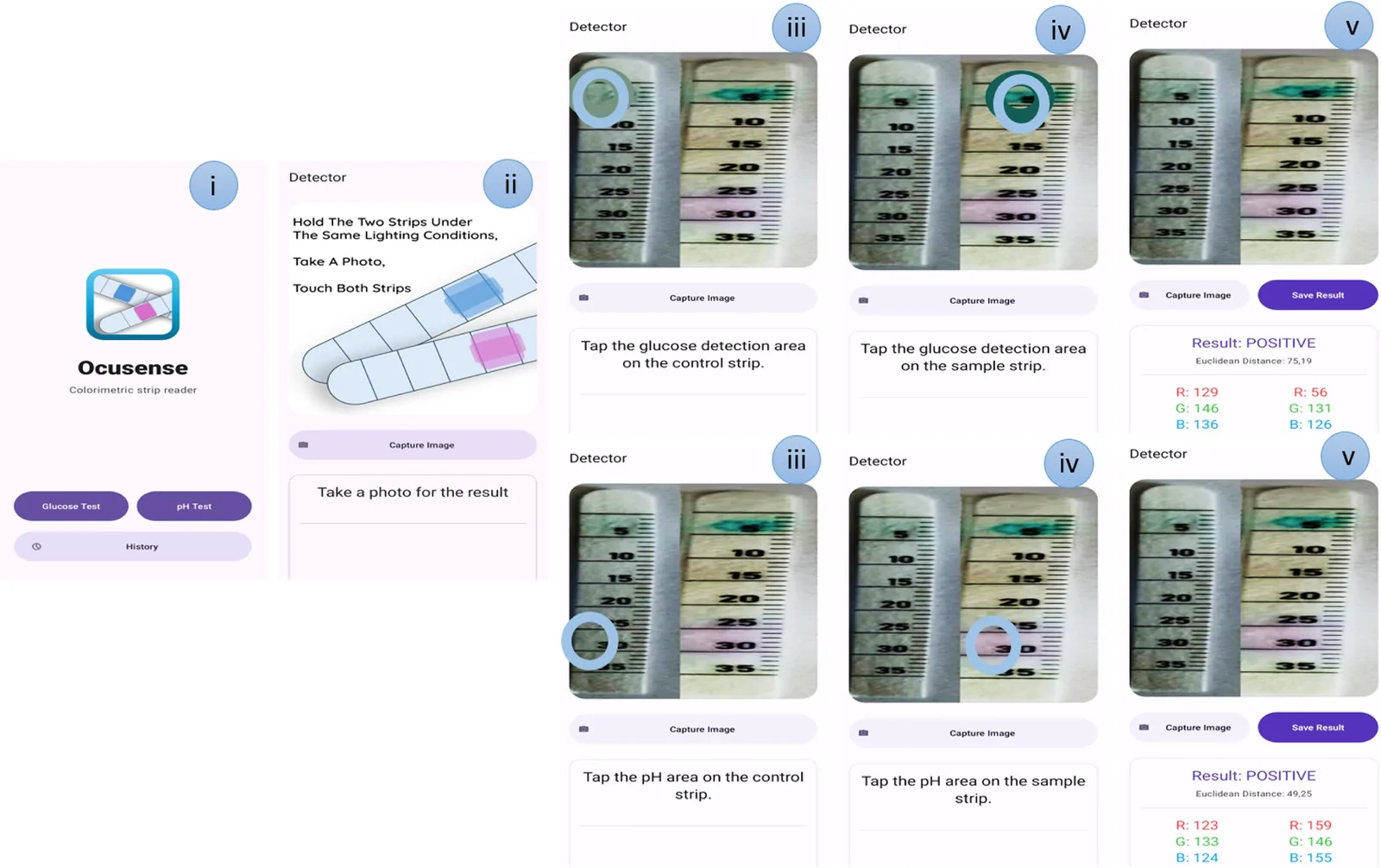
Interfaces of smartphone applications.

## 4 Conclusions

We have developed a simple and rapid dual-functional colorimetric Schirmer strip capable of simultaneously detecting tear glucose and pH using HRP–GOx NF and anthocyanin molecules. The paper-based platform enabled visual glucose detection within 30 s with a detection limit of 0.5 mM, while maintaining high selectivity and providing distinct pH-dependent color responses. The sensor with smartphone-assisted RGB analysis improved the objectivity, portability and quantitative analysis of the assay. The integration of eye tear collection and dual-analyte sensing into a single disposable strip represents a practical strategy for noninvasive POC diagnostics. Although further validation using clinical tear samples is required, this platform provides a promising potential for future tear-based biosensing applications in diabetes monitoring and ocular health evaluation.

## Supplementary Material



## Data Availability

The data supporting this article has been included as part of the Supporting Information (SI).

## Notes

The immobilization of HRP-GOx NFs achieved using the postmodification and immersion methods; data collected on anthocyanin at different concentrations and the Schirmer flow test (PDF)
