# Supplementary material for: A Smartphone-Assisted Schirmer Strip for Rapid Dual Colorimetric Sensing of Tear Glucose and pH Level
Source: ACS Omega. 2026 Oct 13;11(42):65895–904. doi: 10.1021/acsomega.6c10289 (PMC13717788; doi:10.1021/acsomega.6c10289)
Supplement: Supplementary file 1 [file ao6c10289_si_001.pdf]

Supporting Information

A Smartphone-Assisted Schirmer Strip for Rapid Dual Colorimetric Sensing of Tear Glucose and pH Level

Zekeriya Cetinkaya[1], Nimet Temur[2], Umut Gorkem Kocabas[3], Sadik Kucukgunay[4], Humeyra Aslaner[5], Ismail Ocsoy[2*]

[1] Department of Ophthalmology, Kayseri State Hospital, Kayseri 38010, Turkey

[2] Department of Analytical Chemistry, Faculty of Pharmacy, Erciyes University, Kayseri 38039, Turkey

[3] Biomedical Engineering, Faculty of Electrical and Electronics Engineering, Yıldız Technical University, 34220 Esenler, Istanbul

[4] Department of Medical Pharmacology, Faculty of Medicine, Kırşehir Ahi Evran University, Kirsehir 40100, Turkey

[5] Department of Family Medicine, Kayseri Faculty of Medicine, University of Health Sciences, Kayseri 38039, Turkey

Correspond Author: Ismail Ocsoy, ismailocsoy66@gmail.com

RESULTS AND DISCUSSION

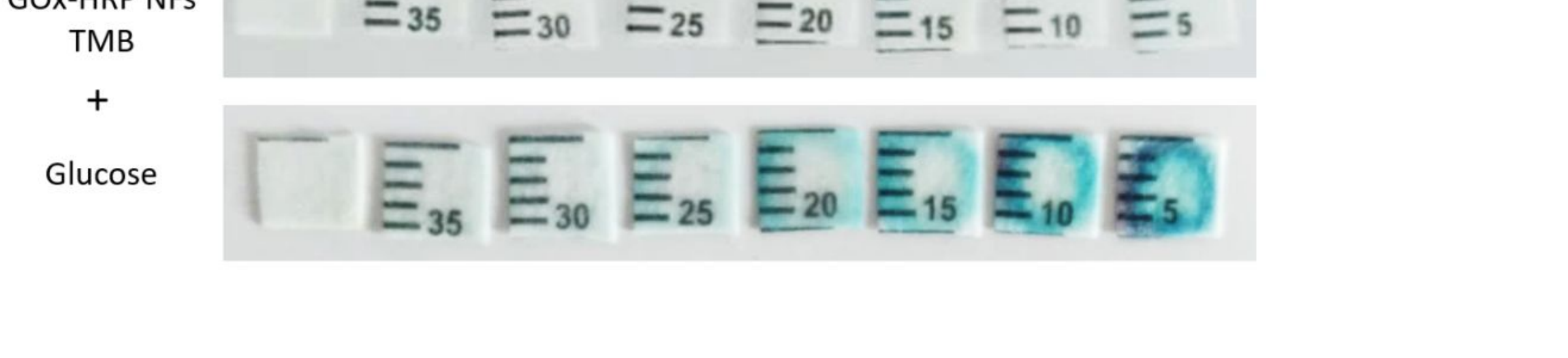

Figure S1. In HRP-GOx@NF synthesized using the post-modification method, a color change occurs upon detection of different glucose concentrations.

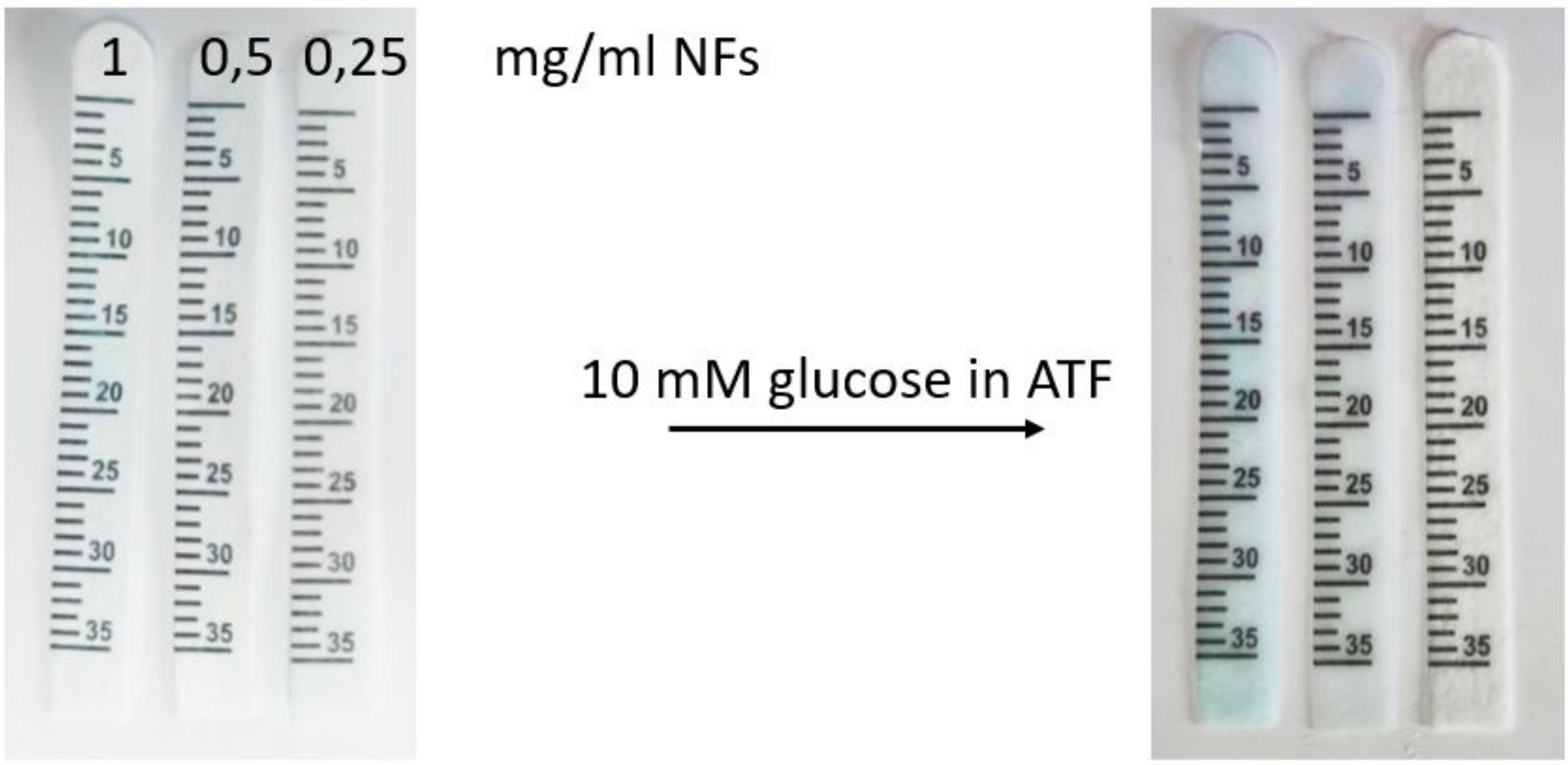

Figure S2. Immobilization of HRP-GOx@NF onto paper: A color change occurs when papers are immersed in different amounts of NF solutions.

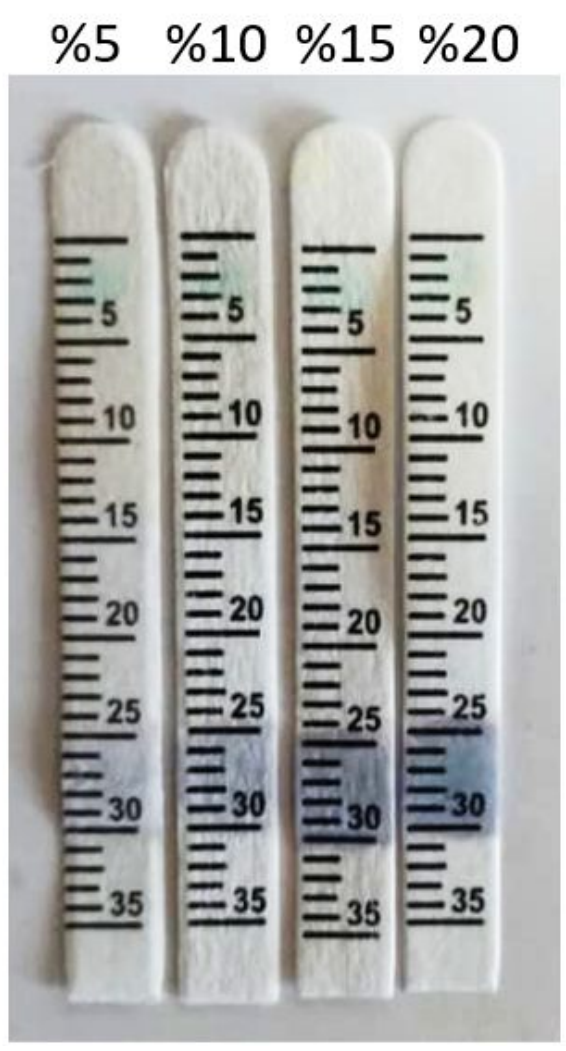

Figure S3. Immobilization of anthocyanins at various concentrations on paper

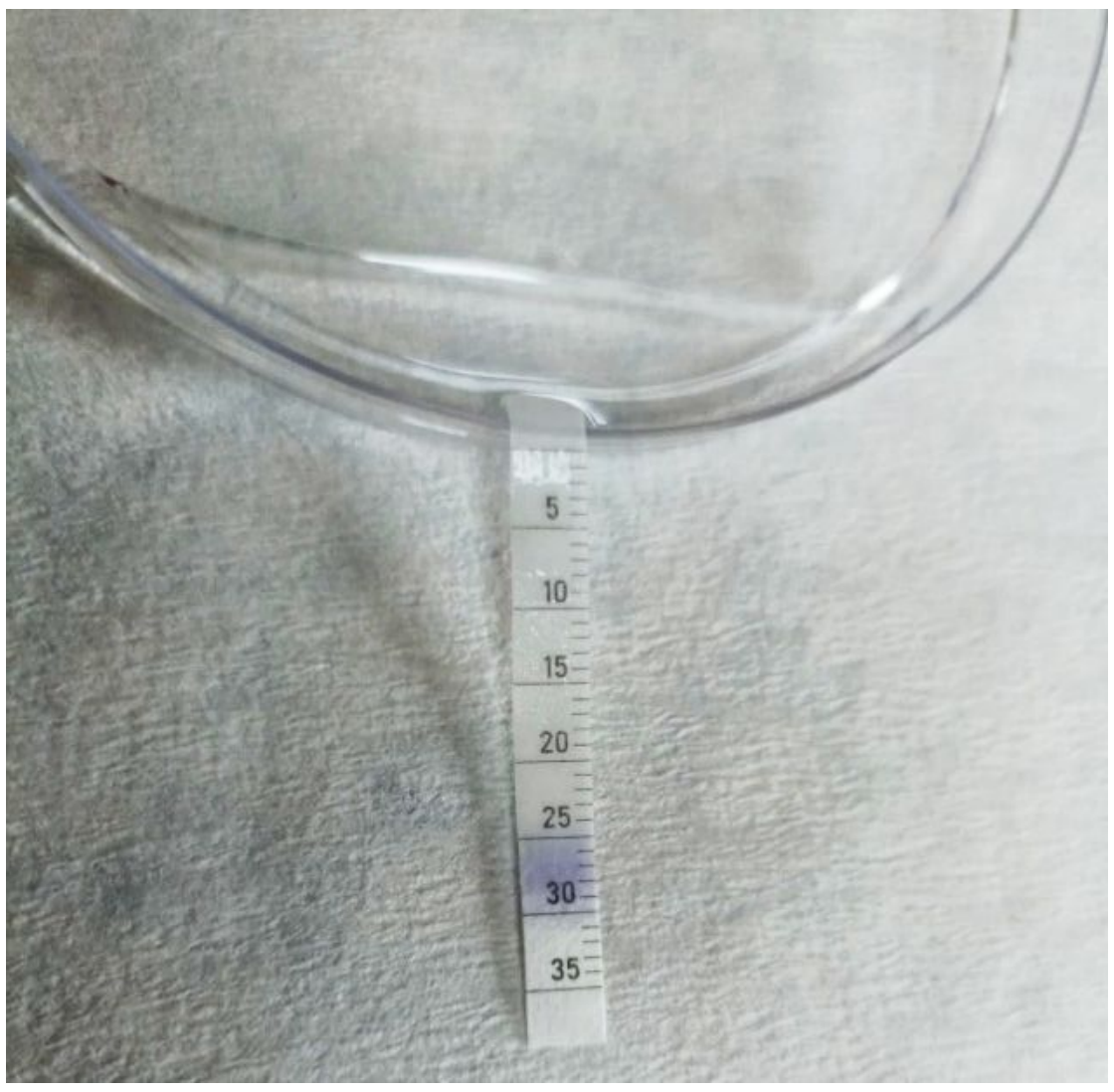

Figure S4. Fluid movement in functionalized Schirmer tests.
